# Progress and challenges in HER2-positive gastroesophageal adenocarcinoma

**DOI:** 10.1186/s13045-019-0737-2

**Published:** 2019-05-17

**Authors:** Dan Zhao, Samuel J. Klempner, Joseph Chao

**Affiliations:** 1 Department of Medical Oncology and Therapeutics Research, City of Hope Comprehensive Cancer Center, Bldg. 51, 1500 E. Duarte Rd, Duarte, CA 91010 USA; 2 The Angeles Clinic and Research Institute, Los Angeles, CA 90025 USA; 3 Samuel Oschin Comprehensive Cancer Institute, Cedars-Sinai Medical Center, Los Angeles, CA 90048 USA

**Keywords:** Gastroesophageal cancer, HER2, Tumor heterogeneity, Trastuzumab, Next-generation sequencing, Genomic profiling, Circulating tumor DNA

## Abstract

HER2 expression remains an important biomarker to guide the addition of the monoclonal antibody trastuzumab to first-line systemic chemotherapy in unresectable, metastatic gastroesophageal adenocarcinomas (GEA). However, in contrast to breast cancer, other HER2-targeted strategies to date have not improved outcomes in this molecular subtype of GEA. Since the initial development of HER2 biomarker testing guidelines, significant spatial intratumoral heterogeneity of HER2 overexpression has been recognized as a major characteristic of this disease. In this review, we aim to survey the seminal positive and negative trials investigating HER2-targeted agents for GEA. We also highlight emerging data on the genomic and temporal heterogeneity of molecular resistance alterations that have yielded further insight into the heterogeneity of therapeutic responses. We conclude with an overview of promising novel agents and strategies which may refine the therapeutic landscape.

## Introduction

Gastric cancer is the fifth most common cancer worldwide and accounts for 6.8% of all cancers excluding non-melanoma skin cancer, and the third most common cause of cancer-specific mortality worldwide according to the latest WHO data [1]. In the USA, gastric cancer represents 1.5% of all new cancers with estimated new cases to be 26,240 and estimated deaths to be 10,800 in 2018 [2]. Despite the trend of decreasing incidence and mortality, the cost and healthcare burden related to gastric cancer increased significantly [3, 4]. Gastric cancer is often diagnosed at an advanced stage, defined as unresectable locoregional or metastatic disease, which has very poor prognosis with 5-year survival not exceeding 5–20%. Systemic chemotherapy remains the mainstay of first-line therapy, with two or three drug combinations of a fluoropyrimidine and a platinum compound, as well as docetaxel and irinotecan being widely used [5, 6]. Approved molecularly targeted therapies for gastric cancer include human epidermal growth factor 2 (HER2)-positive tumors treated with trastuzumab in combination with chemotherapy in the first line and the vascular endothelial growth factor receptor-2 (VEGFR2) inhibitor ramucirumab alone or in combination with paclitaxel in the second line [7]. Apatinib, which is a tyrosine kinase inhibitor that targets VEGFR2, has been approved in China (but not in the USA) for treatment-refractory late-stage gastric cancer [8]. More recently, immune checkpoint inhibitors such as nivolumab (approved in Japan but not in the USA) and pembrolizumab have entered into the treatment armamentarium of systemic therapies for this disease [9, 10]. In February 2019, the US FDA approved trifluridine/tipiracil (TAS-102) for metastatic gastric or gastroesophageal junction adenocarcinoma treated with at least two lines of therapy including HER2-targeted therapy [11]. Hereby, we summarize the current status of HER2-targeted therapies in gastric cancer and emerging data providing further insight into the molecular heterogeneity of this disease.

## Current recommendations for HER2 testing in gastroesophageal cancer

HER2 (also known as erythroblastosis oncogene B2, ERBB2) belongs to the epidermal growth factor receptor (EGFR) family. It is a proto-oncogene whose protein product is a membrane-bound tyrosine kinase receptor which promotes cell proliferation and cancer development upon activation [12]. HER2 can homodimerize or heterodimerize with other EGFR family receptors, such as HER1 (EGFR), HER3, and HER4 to initiate signal transduction of cellular growth pathways [13]. Testing HER2 overexpression using immunohistochemistry (IHC) and fluorescence in situ hybridization (FISH) or other in situ hybridization methods is recommended for all patients with inoperable locally advanced, recurrent, or metastatic gastric adenocarcinoma, based on the guidelines of the American Society of Clinical Oncology (ASCO), the College of American Pathologists (CAP), and the American Society for Clinical Pathology (ASCP) [14]. Patients with positive biomarker results are subsequently candidates for the addition of the anti-HER2 therapeutic monoclonal antibody trastuzumab, which targets the extracellular domain (ECD) of HER2, to frontline chemotherapy [15]. In currently recommended testing algorithms, HER2 status should be tested by IHC first. Positive (IHC 3+) or negative (IHC 0 or 1+) HER2 IHC results do not mandate further in situ hybridization testing. In cases with 2+ expression by IHC (i.e., being equivocal, weak to moderate complete or basolateral or lateral membranous reactivity in ≥ 10% of cancer cells), then in situ hybridization cutoffs using either the HER2/CEP17 (centromeric region of chromosome 17) ratio or copy number–based assessment can be used to delineate final HER2 status. Specifically, in situ hybridization test results of HER2/CEP17 ratio ≥ 2 or an average HER2 copy number ≥ 6.0 signals/cell are considered positive [14]. The rates of HER2 positivity vary by Lauren histologic subtype and primary tumor location (gastroesophageal junction vs gastric body and distal stomach). For instance, in the Trastuzumab for Gastric Cancer (ToGA) trial, the overall HER2 positivity rate was 22.1%, similar between European (23.6%) and Asian patients (23.9%), but higher in Lauren intestinal (31.8%) vs diffuse subtype tumors (6.1%). Of note, gastroesophageal junction (GEJ) tumors had higher HER2 positivity rates (32.2%) than distal or gastric body tumors (21.4%) [16]. Heterogeneity of HER2 IHC staining was noted in around 50% of cases as demonstrated by variability in intratumoral HER2 overexpression (i.e., ≤ 30% of tumor cells exhibiting staining) with greater heterogeneity in lower IHC staining categories [16].

## First-line HER2-targeted trials

In 2010, the phase 3 ToGA trial first demonstrated the benefit of adding trastuzumab to first-line chemotherapy in patients with HER2-positive (IHC 3+ or FISH amplified HER2/CEP17 ≥ 2) locally advanced, recurrent, or metastatic gastric or GEJ adenocarcinoma [17]. Five hundred ninety-four patients were randomized to either trastuzumab combined with chemotherapy (fluorouracil or capecitabine plus cisplatin) or chemotherapy alone. Three thousand six hundred sixty-five patients needed to be screened to obtain a sufficient study population to power the trial. Most patients had distal or gastric body cancers (80% in both groups). The study met its primary endpoint of significantly improving median overall survival (OS) with trastuzumab plus chemotherapy vs chemotherapy alone in the intent-to-treat population (13.8 vs 11.1 months, HR, 0.74; *P* = 0.0046). In a post hoc subgroup analysis, the OS benefit of adding trastuzumab appeared limited to patients whose tumors were HER2 IHC 2+ and FISH positive or IHC 3+ (*n* = 446, 16.0 vs 11.8 months, HR, 0.65; 95% CI 0.51–0.83), but not in cases where tumors were IHC 0 or 1+ despite being FISH positive (*n* = 131, 10 vs 8.7 months, HR = 1.07). Efforts to optimize trastuzumab dosing in HER2-positive metastatic gastric and GEJ cancer were explored in the phase 3 HELOISE trial which tested standard vs higher dosing of trastuzumab (8 mg/kg loading dose, followed by 6 mg/kg vs 10 mg/kg every 3 weeks) with chemotherapy (cisplatin and capecitabine) [18]. However, no significant difference was detected for median OS between the two doses (12.5 months in the trastuzumab 8 mg/kg loading dose followed by 6 mg/kg arm vs 10.6 months in the trastuzumab 10 mg/kg every 3 weeks, *P* = 0.2401). Safety was also comparable between both arms. With the HELOISE trial failing to demonstrate a clinical benefit with higher dosing, trastuzumab at a loading dose of 8 mg/kg followed by 6 mg/kg maintenance dose every 3 weeks with chemotherapy (consistent with the ToGA trial) remains the standard of care for the first-line treatment of HER2-positive metastatic gastric or GEJ adenocarcinoma.

Lapatinib is a small molecule tyrosine kinase inhibitor (TKI) which blocks both HER1 (EGFR) and HER2 signaling. It is approved as the second-line treatment of HER2-positive breast cancer. However, unlike breast cancer, the studies of lapatinib in HER2-positive gastric cancer have not demonstrated the same magnitude of efficacy. The phase 3 LOGiC trial compared lapatinib in combination with capecitabine plus oxaliplatin versus capecitabine plus oxaliplatin alone in HER2-positive advanced or metastatic esophageal, gastric, or GEJ adenocarcinoma [19]. The results exhibited no significant difference in median OS (12.2 vs 10.5 months, HR = 0.91; 95% CI 0.73–1.12, *P* = 0.3492) and median PFS (6.0 vs 5.4 months, *P* = 0.0381), although overall response rate (ORR) was significantly higher in the lapatinib arm (53% vs 39%, *P* = 0.0031). There was no correlation between HER2 IHC status with OS, but the pre-planned subgroup analyses showed Asian and younger patients had longer OS. The lapatinib group exhibited more toxicities especially higher rates of diarrhea (58% vs 29% all grades), with 12% in the lapatinib arm and 3% in the placebo arm having grade ≥ 3 diarrhea [19]. No pharmacokinetics were conducted in this trial to ascertain if gastric cancer patients with prior partial or total gastrectomy have impacted lapatinib absorption, though subset analyses suggested more benefit with the addition of lapatinib to those patients with an intact pylorus (HR, 0.90; 95% CI 0.63–1.01) vs those without (HR, 1.06; 95% CI 0.67–1.68). This trial used central laboratory confirmation of HER2 amplification, and retrospective analysis showed higher HER2 amplification was associated with better PFS, especially in Asian patients (< 60 years old) who had 5.01–10.0 and ≥ 10 fold HER2 amplification treated with lapatinib [20]. The level of amplification may reflect the dependency and “potency” of the driver, thus affect the responses to TKIs. Regardless, with this trial not meeting its primary endpoint, lapatinib has not entered into the frontline treatment armamentarium for advanced HER2-positive gastroesophageal cancer.

Pertuzumab is a monoclonal humanized immunoglobulin (Ig) G1 antibody that targets the heterodimerization domain of HER2, which prevents the heterodimerization of the HER2/HER3 receptors and subsequent downstream signaling [21]. It was approved in HER2-positive breast cancer for combination therapy with trastuzumab and chemotherapy. A phase 2a study suggested the preliminary activity of first-line pertuzumab in combination with trastuzumab, capecitabine, and cisplatin in HER2-positive advanced gastric cancer patients [22]. In this study, patients received pertuzumab 840 mg at cycle 1 and then 420 mg every 3 weeks (q3w) for cycles 2–6 (arm A) or pertuzumab 840 mg q3w for all 6 cycles (arm B). Meanwhile, patients also received trastuzumab, cisplatin, and capecitabine for 6 cycles, then trastuzumab q3w until disease progression or unmanageable toxicity. ORRs for patients treated with pertuzumab plus trastuzumab and chemotherapy were 86% in arm A and 55% in arm B. Based on the available pharmacokinetic and safety data, the 840-mg q3w pertuzumab dose was tested in the phase 3 JACOB trial [23]. In this trial, 780 patients with metastatic gastric or GEJ cancer were assigned to pertuzumab, trastuzumab, and chemotherapy or placebo with trastuzumab and chemotherapy. At a median follow-up of 24.4 months in the pertuzumab group and 25.0 months in the placebo group, no statistically significant difference was found in the primary endpoint of OS (17.5 vs 14.2 months, HR = 0.84, *P* = 0.057), although there was a significant increase in median PFS (8.5 vs 7.0 months, HR = 0.73, 95% CI 0.62–0.86). Forty-five percent of the pertuzumab group patients and 39% of the placebo group patients experienced serious adverse events, with 13% vs 6%, respectively, exhibiting grade ≥ 3 diarrhea. As the JACOB trial did not meet its primary endpoint, pertuzumab has still not entered into routine clinical practice for metastatic gastroesophageal cancer as it has for breast cancer.

## Second-line HER2-targeted trials

Given it appears to be an effective strategy in breast cancer, a continuation of anti-HER2 therapy beyond the first progression on trastuzumab has been an active area of investigation for patients with HER2-positive advanced gastric cancer. A retrospective multicenter study analyzed outcomes of second-line chemotherapy with or without trastuzumab after initial progression on platinum-based chemotherapy with trastuzumab for patients with HER2-positive advanced gastric adenocarcinoma [24]. Continuing (*n* = 39) trastuzumab beyond progression was associated with significantly longer median PFS (4.4 vs 2.3 months; *P* = 0.002) and OS (12.6 vs 6.1 months; *P* = 0.001) compared with discontinuation (*n* = 65) of trastuzumab beyond progression. The benefits of continuing trastuzumab beyond progression remained significant in the multivariate analyses of ECOG performance status, number of metastatic sites, and measurable disease, with observation of longer median PFS (HR, 0.56; *P* = 0.01) as well as OS (HR, 0.47; *P* = 0.004) with this strategy. Given the potential for confounding from retrospective analyses, evidence from prospective trials was still needed before the continuation of trastuzumab could be recommended in routine clinical care.

The first foray prospectively testing this strategy was a study of trastuzumab emtansine (T-DM1), another HER2-targeted therapy FDA-approved in breast cancer, which is an antibody-drug conjugate that links trastuzumab with the cytotoxic agent DM1 (microtubule inhibitor, a maytansine derivative) [25, 26]. Similar to trastuzumab, T-DM1 binds to HER2 and inhibits the downstream signaling pathway and induces antibody-dependent cellular cytotoxicity (ADCC). In addition, after internalization of the HER2-T-DM1 complex, the microtubule inhibitor DM1 payload is released into tumor cells by lysosomal degradation leading to mitotic arrest and apoptosis [27]. As shown in the EMILIA and TH3RESA trials in previously treated HER2-positive advanced breast cancer patients, T-DM1 prolonged PFS and OS with less toxicity compared to chemotherapy in HER2-positive advanced breast cancer [26, 28, 29]. To test the role of T-DM1 in HER2-positive advanced gastric cancer progressed during or after first-line trastuzumab-containing therapy, the GATSBY trial was conducted, which was a randomized phase 2/3 study performed at 107 centers in 28 countries [30]. The first stage of the trial assigned patients (2:2:1) to intravenous T-DM1 (3.6 mg/kg every 3 weeks or 2.4 mg/kg weekly) or the physician’s choice of a taxane (intravenous docetaxel 75 mg/m^2^ every 3 weeks or intravenous paclitaxel 80 mg/m^2^ weekly). Subsequently, after the interim analysis, the independent data monitoring committee selected T-DM1 2.4 mg/kg weekly to proceed to stage 2, for which patients were assigned (2:1) to either the T-DM1 or the abovementioned taxane. The pharmacokinetic profiling demonstrated the 2.4 mg/kg weekly regimen provided twice the dose intensity compared to the typical dose used in HER2-positive metastatic breast cancer (3.6 mg/kg every 3 weeks) without concerning new safety signals [31]. For the analysis of stage 2 results, the median follow-up was 17.5 months for the T-DM1 2.4 mg/kg weekly group (*n* = 224) and 15.4 months in the taxane group (*n* = 111). Despite higher dose intensity achieved when compared to standard dosing in breast cancer, there was no difference between T-DM1 2.4 mg/kg weekly and single-agent taxane in the primary endpoint of median OS (7.9 vs 8.6 months, HR, 1.15; 95% CI 0.87–1.51, one-sided *P* = 0.86). The T-DM1 2.4 mg/kg group did demonstrate a lower incidence of grade 3 or higher adverse events (60% vs 70%), though similar serious adverse events (29% vs 28%) and adverse events leading to treatment discontinuation (14% vs 14%) or death (4% vs 4%) compared to the taxane group [30]. Thus, the GATSBY trial concluded that T-DM1 was not superior to taxane for previously treated, HER2-positive advanced gastric cancer. This study did require central laboratory testing of a primary or metastatic tumor to confirm positivity for HER2. However, HER2 testing was not mandated of a new tumor biopsy prior to trial entry, and thus, the temporal heterogeneity in HER2 expression (i.e., HER2 loss) after progression on first-line therapy was not captured. As such, any positive signal of anti-tumor efficacy in the T-DM1 arm of the trial may have been diluted by the lack of activity in patients whose tumors no longer retained HER2 positivity.

Lapatinib was also tested in the second-line setting for HER2-positive gastric cancer. The phase 3 TyTAN trial compared lapatinib 1500 mg daily plus weekly paclitaxel 80 mg/m^2^ or paclitaxel alone as the second-line treatment in patients with HER2 FISH-positive advanced gastric cancer. Due to the time period of trial enrollment with initial reporting of ToGA results, only a very small minority (~ 6%) of patients had received trastuzumab-containing first-line therapy resulting in a predominantly anti-HER2 treatment-naïve population. Despite this, there was no significant difference found in the median OS (11.0 vs 8.9 months, HR, 0.84; 95% CI 0.64–1.11; *P* = 0.1044) or median PFS (5.4 vs 4.4 months, HR, 0.85; 95% CI 0.63–1.13; *P* = 0.2441) between the lapatinib plus paclitaxel vs the paclitaxel alone groups. ORR was higher with lapatinib plus paclitaxel vs paclitaxel alone (27% vs 9%; OR, 3.85; 95% CI 1.80–8.87; *P* < 0.001). Lapatinib plus paclitaxel demonstrated better efficacy in IHC 3+ compared to IHC 0/1+ and 2+ patients and in Chinese patients compared with Japanese patients [32]. In IHC 3+ patients, OS (HR, 0.59; 95% CI 0.37–0.93; *P* = 0.0176) as well as PFS (HR, 0.54; 95% CI 0.33–0.90; *P* = 0.0101) appeared improved when adding lapatinib to paclitaxel. However, no OS or PFS benefits were observed with adding lapatinib to paclitaxel in IHC 0/1+ or IHC 2+ patients. The results of TyTAN seem to suggest that some benefit of second-line anti-HER2 therapy can still be achieved if there can be better enrichment of patients with stronger HER2 overexpression of their tumors.

True testing of continuing HER2 inhibition with trastuzumab and changing of the chemotherapy backbone after progression on first-line therapy was recently examined in the prospective randomized phase 2 T-ACT trial [33]. This study compared weekly paclitaxel with or without trastuzumab beyond progression in patients with HER2-positive advanced gastric or GEJ adenocarcinoma refractory to fluoropyrimidine, platinum, and trastuzumab first-line therapy. In the T-ACT trial, 45 patients were randomized to paclitaxel 80 mg/m^2^ on days 1, 8, and 15 every 4 weeks and 44 patients were assigned to paclitaxel plus trastuzumab (8 mg/kg loading dose with 6 mg/kg every 3 weeks). As reported at the 2018 ASCO annual meeting, there was no difference in the primary endpoint of median PFS (3.19 vs 3.68 months, *P* = 0.334) or secondary endpoint of median OS (9.95 vs 10.20 months, *P* = 0.199). The study investigators did collect new tumor biopsy samples among 16 patients at the timing of after progression on first-line trastuzumab-containing therapy and prior to enrollment on the T-ACT trial. While this represented only a small proportion of their patient population, what was compelling is the authors observed that 11 of the 16 patients (69%) lost HER2 positivity as ascertained by standard IHC and FISH testing. To date, this represents one of the highest reported rates of HER2 loss in the context of patients being enrolled onto a second-line trial of HER2-directed therapy and highlighting a role for repeat ascertainment of HER2 status.

## Tumor heterogeneity as a challenge for anti-HER2 therapy in gastroesophageal cancer

Table 1 summarizes the landmark trials for HER2-positive gastric cancer. In contrast to HER2-positive breast cancer, approved HER2-directed therapies remain limited for gastric cancer, as exemplified by the abovementioned negative trials with lapatinib, pertuzumab, and T-DM1. Several mechanisms of resistance to anti-HER2 therapies in gastric cancer have been proposed pertaining to the molecular heterogeneity of these tumors, both *inter*-patient and *intra*tumorally. Inter-patient molecular heterogeneity has been exemplified by multi-platform, high-throughput sequencing efforts such as those put forth by the Asian Cancer Research Group (ACRG) and The Cancer Genome Atlas (TCGA) [34–36]. The TCGA analysis has posited four major molecular subgroups of gastric cancer: microsatellite instable (MSI), Epstein-Barr virus (EBV) associated, chromosomally instable (CIN), and genomically stable (GS) tumors. Likewise, the ACRG effort has distinguished four molecular subtypes of gastric cancer: MSI, microsatellite stable (MSS)/TP53 active, MSS/TP53 inactive (i.e., mutated), and MSS/mesenchymal like (EMT). Of interest is that *HER2* gene amplification corresponding to HER2 overexpression is associated with the CIN subgroup by TCGA classification and the MSS/TP53 inactive subtype by the ACRG categorization. Both molecular subgroups share the commonality of widespread genomic instability characterized by mutation in the p53 tumor suppressor, which likely facilitates significant copy number variations of major oncogenic drivers including HER2.

**Table 1:** Landmark clinical trials of HER2-positive gastric cancer

Trials	Patients	Line of therapy	Region	Phase	Study arms	Results
ToGA [17]	HER2-positive, locally advanced, recurrent or metastatic gastric and GEJ adenocarcinoma	1st	Global	3	Trastuzumab plus chemotherapy (fluorouracil or capecitabine and cisplatin) vs chemotherapy alone	Improvement of median OS with trastuzumab plus chemotherapy (13.8 vs 11 months, *P* = 0.0046)
HELOISE [18]	HER2-positive metastatic gastric cancer and GEJ cancer	1st	Global	3	Trastuzumab (8 mg/kg loading dose, followed by 6 mg/kg VS 10 mg/kg every 3 weeks) plus cisplatin (80 mg/m^2^ on day 1) and capecitabine (800 mg/m^2^twice daily on days 1–14)	No difference in median OS 12.5 vs 10.6 months (stratified HR, 1.24; 95% CI 0.86–1.78; *P* = 0.2401)
TyTAN [32]	HER2 FISH-positive IHC 3+ advanced gastric cancer	2nd	Asia	3	Lapatinib plus weekly paclitaxel vs paclitaxel alone	No difference in median OS (11.0 vs 8.9 months, *P* = 0.1044) nor median PFS (5.4 vs 4.4 months)
LOGIC [19]	HER2-positive advanced or metastatic esophageal, gastric or GEJ adenocarcinoma	1st	Asia	3	Lapatinib with capecitabine plus oxaliplatin vs capecitabine plus oxaliplatin	No difference in median OS (12.2 vs 10.5 months, HR, 0.91; 95% CI 0.73–1.12, *P* = 0.3492) and median PFS (6.0 vs 5.4 months, *P* = 0.0381).
JACOB [23]	HER2-positive metastatic gastric cancer or GEJ cancer	1st	Global	3	Pertuzumab, trastuzumab, and chemotherapy vs trastuzumab and chemotherapy	No difference in median OS (17.5 vs 14.2 months, *P* = 0.057)
GATSBY [30]	HER2-positive gastric cancer	2nd	Global	2/3	IV TD-M1(2.4 mg/kg weekly) vs taxane (docetaxel 75 mg/m^2^every 3 weeks or paclitaxel 80 mg/m^2^ weekly)	No difference in median OS (7.9 vs 8.6 months, *P* = 0.86).
T-ACT [33]	HER2-positive advanced gastric or GEJ adenocarcinoma	2nd	Japan	2	Paclitaxel 80 mg/m^2^ on days 1, 8, and 15 every 4 weeks vs paclitaxel plus trastuzumab	No difference in median PFS (3.19 vs 3.68 months, *P* = 0.334) and median OS (9.95 vs 10.20 months, *P* = 0.199).

Intratumoral heterogeneity has been observed as early as studies developing initial HER2 IHC testing, with the heterogeneity of tumor cell HER2 IHC staining observed to be greater in gastroesophageal compared to breast adenocarcinomas, with staining also more often seen in a basolateral and less complete membranous pattern than breast cancer [37]. In sum, molecular profiling studies have demonstrated HER2-positive gastric cancer is not a homogenous disease, and differing genetic alterations can co-exist with HER2 in different patients’ tumors as well as intratumorally where subclones of tumor cells can harbor differing molecular characteristics driving multiple redundant signaling pathways. An abstract presented by Klempner et al. analyzed comprehensive genomic profiling data of 2245 GEJ and 1883 distal or gastric body adenocarcinomas (GC) using the targeted next-generation sequencing (NGS) FoundationOne platform and identified 395 HER2-amplified (HER2amp) GEJ (18%) and 132 HER2-amp GC (7.0%) cases. PIK3CA genomic alterations and MET amplification was observed in around 9% and 5% of both HER2 amplified and non-HER2 amplified EGC cases [38]. However, co-amplification of cell-cycle mediators CDK6 (11% vs 6.8%) and CCNE1 (19% vs 7.1%), MYC (16% vs 9.8%), and deleterious SMAD4 genomic alterations (9.7% vs 5.5%) were enriched in cases with versus without HER2 amplification. These results indicate from a large real-world clinical practice dataset the extent of baseline tumoral heterogeneity present at initial gastroesophageal cancer presentation. Due to the dynamic and unstable nature of cancer’s genome, as well as intratumoral heterogeneity, and inevitable clonal evolution, change of HER2 status may not be uncommon in gastric cancer and likely contributes as a major mechanism of acquired resistance to anti-HER2 treatment. As mentioned previously, the T-ACT investigators observed a rate as high as 69% of HER2 loss between the first- and second-line treatment with traditional IHC and FISH testing methods [33]. Janjigian et al. also reported an analysis of 44 patients with post-trastuzumab tumor tissue samples subjected to targeted NGS using the MSK-IMPACT panel and observed loss of HER2 amplification in 7 (14%) of the tumors [39]. In addition, other secondary alterations that putatively predict for resistance to anti-HER2 therapy were captured and appeared to be enriched at greater frequency compared to pre-treatment samples including exon 16 deletion of the HER2 gene and mutations in KRAS and PI3K signaling pathways. Also of interest, among 50 patient samples in which HER2 amplification at pre-treatment baseline was quantitated by targeted NGS in this study, they observed the longest median PFS (24.3 months) to first-line trastuzumab in patients with the highest quartile of HER2 amplification and significantly shorter median PFS (8.4 months) in patients with detected co-alterations in RTK-RAS-PI3K/AKT pathway genes. A separate study of both breast and gastroesophageal cancers using the MSK-IMPACT NGS platform showed an overall concordance of 98.4% with IHC/FISH testing for HER2 status, and discrepancies were attributed to low tumor content and intratumoral HER2 heterogeneity, suggesting NGS can be reliable for HER2 testing [40]. As such, NGS analyses to discern baseline inter-patient tumoral genomic heterogeneity may further refine predicted benefit of anti-HER2 therapy.

With the advancement of NGS and liquid biopsy circulating tumor DNA (ctDNA) assays, these technologies are being increasingly studied in efforts to move beyond or supplement traditional HER2 IHC and FISH testing in enriching patients for HER2-targeted therapy. In a large-scale study of 21,807 treated late-stage cancer patients of 50 differing solid tumor types, including 328 gastric cancer patients, somatic mutations in ctDNA were detectable in 85% of all patients tested using the Guardant360 targeted panel of 70 cancer genes [41]. Another study performed ctDNA profiling using the FoundationACT platform of 62 genes among 417 gastrointestinal carcinoma patients (8.9% were gastric adenocarcinoma), which demonstrated ctDNA being detectable in 344 of 417 samples (82%), with 89% (306/344) of these samples having more than 1 reportable genomic alterations detected [42]. CtDNA sequencing routinely detected additional alterations not found in matched tumor tissue NGS, findings of which can be consistent with intrapatient tumoral heterogeneity. Kim et al. also evaluated in a prospective fashion tumor tissue NGS and ctDNA through parallel biomarker studies within a single-arm phase 2 study of lapatinib with capecitabine and oxaliplatin in advanced HER2-positive gastric adenocarcinoma as the first-line therapy [43]. Among 16 tumor specimens with sufficient tissue quality for NGS, CCNE1 amplification was the most common co-occurring copy number alteration, found in 40% of HER2-positive tumors and trended to lack of response to HER2-targeted therapy (66.7% of non-responders had CCNE1 amplification versus 22.2% of responders, *P* = 0.08). Compared to patients with low-level HER2 amplification, patients with high-level HER2 amplification by NGS were also more likely to respond to therapy (mean predicted HER2 copy number 24.17 in responders vs 3.3 in non-responders, *P* = 0.02). In an analysis of ctDNA (*n* = 9, 8 assessable) using the Guardant platform, 6 of 8 patients had detectable HER2 copy number amplification in plasma and all 6 of these patients responded to treatment (6/6, 100% response rate). There was an association between temporal changes in plasma-detected genomic alterations and sensitivity and/or resistance to lapatinib-based therapy. Interestingly, 3 of 7 post-progression biopsies of the primary tumor amongst the non-responders demonstrated loss of HER2 overexpression. Follow-up ctDNA profiling at disease progression also demonstrated variability in the emergence of other genomic alterations such as MYC, EGFR, FGFR2, and MET amplification. These findings highlight the likely inter-patient and intrapatient spatial and temporal genomic heterogeneity that occurs when resistance to therapy develops. Sukawa et al. also reported as one of the companion biomarker studies to the T-ACT trial cell-free DNA analyses focused on detection of circulating HER2 gene amplification (cfHER2amp) [44]. Among 68 patients assessable, cfHER2amp was positive in 41 (60%), though the benefit of trastuzumab beyond progression did not appear to correlate to the presence (HR, 0.93, 95% CI 0.49–1.76) or absence (HR 0.81, 95% CI 0.36–1.85) of cfHER2amp. The lack of predictive benefit with a single timepoint of cfHER2amp assessment could argue for the importance of serial sampling and broader gene panel testing to better capture temporal and spatial heterogeneity in elucidating resistance to anti-HER2 therapy.

Due to the emerging picture of tumoral heterogeneity of HER2 positive in particular and gastroesophageal cancers in general, larger datasets will be needed to ascertain if composite testing with traditional IHC/FISH, tumor NGS, and ctDNA, as well as other biomarkers, can enrich for patients that derive the most clinical benefit in HER2-directed strategies. Future trials should collect this data in a prospective fashion as integrated biomarkers with multiple sampling timepoints (i.e., re-biopsy at progression and/or serial liquid biopsies) may best capture spatial and temporal tumor heterogeneity. Repeat biopsies of single metastatic sites will likely be limited by sampling issues but may still complement ctDNA analyses. The optimal sensitivity and specificity of ctDNA testing over time and whether ctDNA is truly reflective of the predominant metastatic tumor burden remain open areas of investigation. Questions also remain if known tumor tissue mutations that are not detected in ctDNA are truly reflective of loss of that particular subclone or an artifact of a “low ctDNA shedding” vs “high ctDNA shedding” tumor. Continuation of HER2-directed therapies likely will only be beneficial for those with retention of HER2 overexpression, though the benefit may be impacted by the mechanism of the agent and ability to overcome resistance brought about by co-alterations such as RAS/PI3K pathway changes. In cases where co-amplifications of oncogenic receptor tyrosine kinases are observed (such as in MET, FGFR2, or EGFR), will combining multiple targeted therapies be necessary at the potential cost of greater toxicity or will sequential targeted therapy strategies be viable enough to eliminate tumor subclones in a sequential fashion? Continued translational analyses and improved preclinical models of intratumoral heterogeneity to study clonal evolution and drug resistance may hopefully elucidate this question.

## Novel anti-HER2 agents and strategies under investigation

**Fig. 1:**
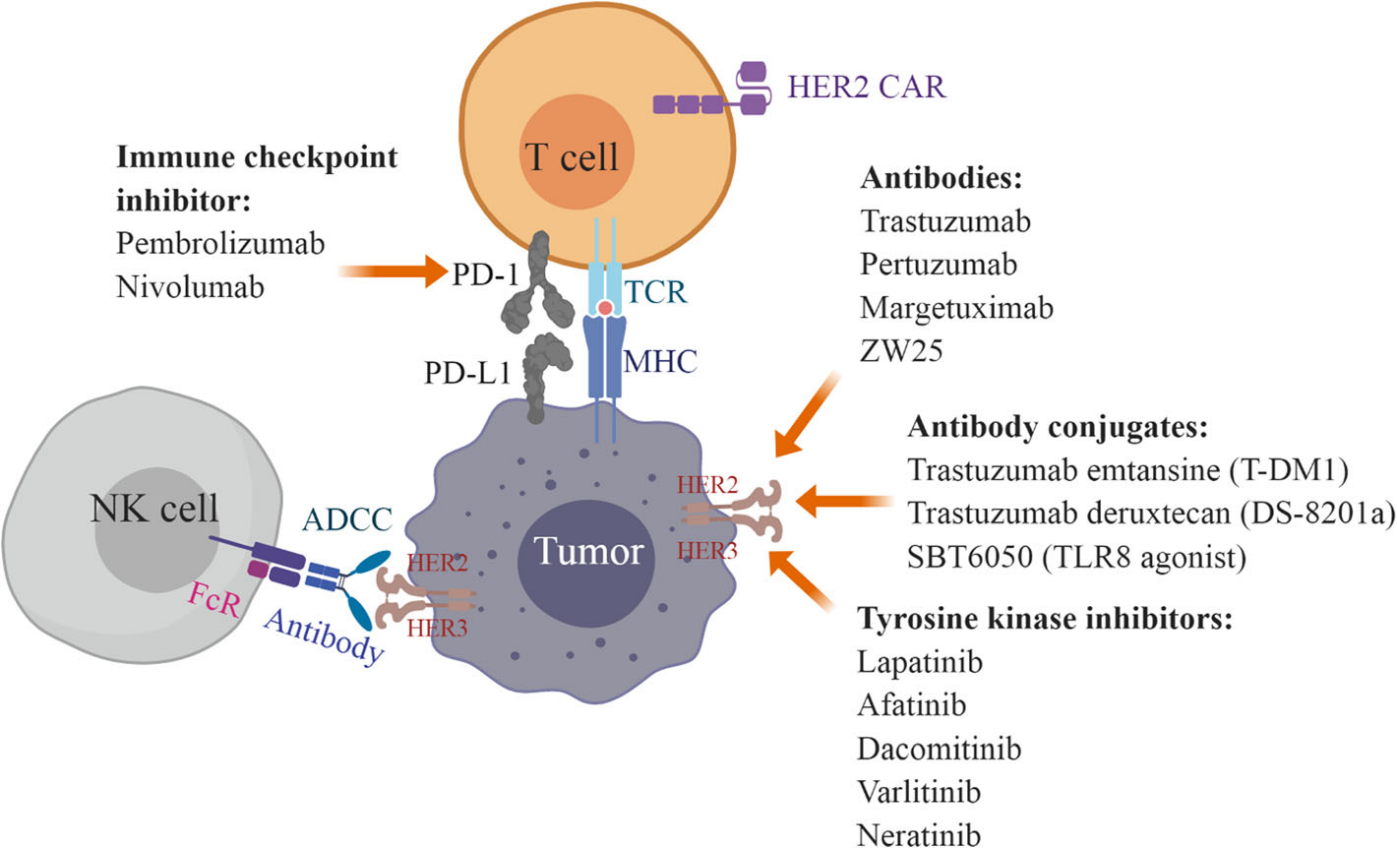
Strategies for targeting HER2-positive gastric cancer. Anti-HER2 antibodies included trastuzumab, pertuzumab, margetuximab, and ZW25. Anti-Her2 antibody conjugates included trastuzumab emtansine (T-DM1), trastuzumab deruxtecan (DS-8201a), and SBT6050 (TLR8 agonist). Tyrosine kinase inhibitors targeting HER2 included lapatinib, afatinib, dacomitinib, varlitinib, and neratinib. Fc receptors (FcR) expressed on NK cell (natural killer cell) bind to antibodies against HER2 and trigger anti-tumor immune response via antibody-dependent cellular cytotoxicity (ADCC). NK cell products in combination with trastuzumab for HER2-positive tumors were under investigation. Immune checkpoint inhibitors target program death 1 (PD-1)/programmed death-ligand 1 (PD-L1), the co-inhibitory signals for T cell antigen receptor (TCR) signaling, to enhance T cell anti-tumor immunity. Chimeric antigen receptor (CAR)-T cells expressing HER2-specific CAR maybe an option for HER2-positive gastric cancer. MHC major histocompatibility complex. The figure was created with Biorender.com.

As summarized in Fig. 1, HER2-targeted strategies in gastric cancer consist of monoclonal antibodies (e.g., trastuzumab, pertuzumab, margetuximab), TKIs (e.g., lapatinib, afatinib, dacomitinib, varlitinib, neratinib), bispecific antibodies (e.g., ZW25), antibody conjugates (e.g., T-DM1, trastuzumab deruxtecan/DS-8201a, SBT6050), and cellular-based therapies using T cells and NK cells [45]. Currently, there are more than 30 ongoing clinical trials testing anti-HER2 therapy in gastric cancer which may inform the treatment landscape in options beyond trastuzumab. Some of the interest includes trastuzumab deruxtecan (DS-8201a), a novel anti-HER2 antibody conjugate [46]. The initial publication of a phase 1 trial in breast and gastric cancer reported no dose-limiting toxicities and a 43% ORR with a 91% disease control rate among 23 patients [47]. Follow-up reporting of this phase 1 trial with the inclusion of expansion cohorts has continued to demonstrate acceptable safety in 241 evaluable patients, though 5 cases of grade 5 interstitial lung disease/pneumonitis were observed and undergoing central adjucation at the time of study reporting [48]. Efficacy however has been compelling even in a heavily pre-treated patient population given among the HER2-positive gastric cancer subgroup (*n* = 44), 43.2% had confirmed RECIST responses with a median PFS of 5.6 months and median duration of response of 7 months. A more intriguing aspect of this agent are preclinical experiments demonstrating significant anti-tumor activity via a bystander effect where HER2 overexpressing cells are recognized by DS-8201a but nearby HER2-negative cells in co-culture also underwent apoptosis [49]. This bystander effect appears unique to DS-8201a as such anti-tumor activity in preclinical experiments was not observed with T-DM1. DS-8201a can thus be a promising agent whose mechanism of action may overcome resistance accounted for by intratumoral heterogeneity of HER2 overexpression and outgrowth of HER2-negative clones.

As listed in Table 2, several TKIs are under investigation for HER2-positive gastroesophageal cancer. Dacomitinib is an irreversible pan-HER inhibitor and was tested in a phase 2 trial of 27 previously treated advanced HER2-positive gastric cancer patients, reporting a modest ORR of 7.4% and a disease control rate (DCR) of 40.7% [50]. Afatinib irreversibly blocks EGFR, HER2, and HER4 and was tested in combination with paclitaxel in solid tumors expressing EGFR or HER2 in a phase 1 study (NCT00809133) [51]. Among 16 patients tested, 5 had partial responses including 1 esophageal cancer patient. Other phase 2 studies of afatinib in combination with paclitaxel remain ongoing (NCT01522768 and NCT02501603). Publication of a recent phase 2 study of afatinib monotherapy or afatinib plus trastuzumab combination therapy in trastuzumab-refractory HER2-positive esophagogastric cancer reported a 10% ORR for afatinib monotherapy (2 out of 20) and 1 partial response with the afatinib/trastuzumab combination and 2 patients (17%) achieving disease control ≥ 4 months [52, 53]. Further highlighting how intratumoral heterogeneity may delineate clinical responses to HER2-targeted strategies, the authors observed a greater reduction in tumor burden to afatinib in cases where *EGFR* and *HER2* gene co-amplification occurred, interestingly in cases where the co-amplification existed within the same clonal tumor cell population confirmed by dual-probe FISH. The exception existed in a case where *MYC* gene amplification also co-existed with EGFR and HER2, thus seemingly mediating resistance to afatinib to a genetic signature otherwise predicting for response. The authors also observed intrapatient tumoral heterogeneity manifesting as concurrent oncogene amplifications existing in differing subclonal populations, exemplified in one case where metastatic progression appeared to be driven by *MET* gene amplification that was not detected in the other non-progressing metastatic sites at post-mortem analysis. Varlitinib (ASLAN001) is a reversible pan-HER inhibitor being studied in gastric, cholangiocarcinoma, breast, and colorectal cancers and is now being examined in a phase 1b/2 trial in combination with mFOLFOX for HER1/HER2 co-expressing gastric cancer (NCT03130790). Neratinib is another irreversible pan-HER inhibitor, recently approved in breast cancer after the phase 3 ExteNET trial demonstrated that 1 year of extended neratinib therapy after adjuvant chemotherapy and trastuzumab for HER2-positive breast cancer improved 5-year invasive disease-free survival (90.2% vs 87.7%, HR, 0.73; *P* = 0.0083) [54]. Neratinib is currently being tested in a basket trial for HER2 mutation-positive or EGFR-amplified solid tumors (SUMMIT/NCT01953926).

**Table 2:** Ongoing phase 2 and 3 trials targeting HER2-positive gastroesophageal cancer

Trials/ID	Treatments	Phase	Patients	Line of therapy	Primary endpoints	Estimated completion date	Region
KEYNOTE-811/NCT03615326	Pembrolizumab/placebo + trastuzumab + chemotherapy	3	HER2-positive advanced gastric cancer or GEJ adenocarcinoma	1st	PFS, OS	June 2023	Global
NCT02954536 [60]	Pembrolizumab, trastuzumab, capecitabine/cisplatin	2	HER2-positive metastatic gastroesophageal cancer	1st	PFS	November 2019	USA
NCT02901301	Pembrolizumab + trastuzumab + capecitabine + cisplatin	1b/2	HER2-positive gastric cancer	1st	RP2D, responses	March 2019	Korea
NCT01522768	Afatinib + paclitaxel	2	Trastuzumab-refractory HER2-positive metastatic or recurrent GEA	> 1	Responses	February 2020	USA
NCT02501603	Afatinib + paclitaxel	2	Advanced/metastatic gastric or GEJ cancer including HER2-positive refractory to trastuzumab	2nd	PFS	December 2018	Korea
DESTINY-Gastric01/NCT03329690	Trastuzumab deruxtecan (DS-8201a) vs physician’s choice	2	HER2-positive advanced gastric or GEJ adenocarcinoma	> 2	Responses	December 2019	Japan
INTEGA/NCT03409848	Trastuzumab + nivolumab + ipilimumab or nivolumab + FOLFOX + trastuzumab	2	HER2-positive locally advanced or metastatic GEA	1st	OS	October 2021	Germany
NCT02689284 [64]	Margetuximab + pembrolizumab	1b/2	Relapsed/refractory advanced HER2-positive GEJ or gastric cancer	>1	MTD, responses	December 2017	Global
NCT02713984	HER2-targeted CAR T cells	1/2	Relapsed or refractory HER2-positive cancer	>1	MTD	September 2018	China
NCT03130790	Varlitinib/placebo + mFOLFOX6	2/3	Co-expression of HER1 and HER2 advanced or metastatic gastric or GEJ adenocarcinoma	1st	Responses, OS	December 2018	Asia
SUMMIT/NCT01953926 [65]	Neratinib monotherapy	2	HER2 mutated cancers excluding colon cancer, lung cancer, breast cancer, and bladder cancer, HER4 mutated cancer	Any	Responses	March 2022	USA
INNOVATION/NCT02205047	Neoadjuvant trastuzumab or trastuzumab + pertuzumab with chemotherapy	2	HER2-positive, resectable gastric cancer	1st	Major pathological response	September 2020	Global

Margetuximab is an Fc-optimized monoclonal antibody against HER2, and ex vivo analyses of patient peripheral blood mononuclear cell samples from a phase 1 study demonstrated margetuximab had enhanced ADCC compared with trastuzumab [55]. Margetuximab is currently under investigation in the phase 3 SOPHIA trial (margetuximab plus chemotherapy vs trastuzumab plus chemotherapy) in HER2-positive metastatic breast cancer. A phase 1/2 trial is also testing margetuximab in advanced gastric cancer in combination with the immune checkpoint inhibitor pembrolizumab [56]. As of the data cutoff analysis of December 4, 2017, the ORR was higher in patients with gastric (*n* = 25) vs GEJ cancer (*n* = 26) (32% vs 4%). Interestingly, the response rate to margetuximab + pembrolizumab in a post-trastuzumab HER2 ctDNA-positive population was 26% (6/23) versus 0% (0/22) in post-trastuzumab HER2 ctDNA-negative patients, while in a small dataset, this finding would support the importance of tracking temporal changes in HER2 overexpression through ctDNA analysis and reserving continuation of anti-HER2 strategies in patients’ tumors that retain the HER2 target.

A bispecific antibody is an engineered protein capable of recognizing and binding two different antigens at the same time. ZW25 is a novel bispecific antibody specifically designed to simultaneously bind two HER2 epitopes, ECD 4 (trastuzumab binding domain) and ECD 2 (pertuzumab binding domain). Promising results from a phase 1 study of single-agent ZW25 were presented at the 2018 ASCO annual meeting. It was well tolerated in heavily pre-treated patients as a single agent, and efficacy was notable with a 56% (5/9) disease control rate in HER2-positive gastroesophageal cancer patients that progressed after prior trastuzumab [57]. Novel anti-HER2 antibody conjugates include SBT6050, which has been designed to carry the Toll-like receptor 8 (TLR8) agonist payload specifically to the tumor microenvironment of HER2 overexpressing cancers [58]. While still in preclinical testing, this novel design may facilitate selective activation of innate and adaptive anti-tumor responses while sparing systemic immune toxicities that have been observed to date with other systemically administered immune cell agonists. Currently, this agent is projected to enter into the clinic in first-in-human trials in 2020.

Immune checkpoint inhibitors targeting the program death 1 (PD-1) and programmed death-ligand 1 (PD-L1) signaling pathway have changed the paradigm of cancer therapy in recent years. The PD-1 inhibitors pembrolizumab and nivolumab have garnered regulatory approval in the USA and Japan, respectively, for third-line therapy of metastatic gastroesophageal adenocarcinoma based on large trials exhibiting therapeutic benefit [9, 10]. It appears rational to combine immune checkpoint inhibitors with monoclonal antibodies such as trastuzumab given ADCC is an important mechanism of anti-tumor activity and preclinical experiments have supported HER2 inhibition enhancing T cell activation [59]. While the previously mentioned margetuximab study has studied this in a refractory population, major interest has arisen in testing this strategy in a treatment-naïve population. Two such ongoing phase 2 trials (NCT02954536 and NCT02901301) are combining pembrolizumab with trastuzumab, fluoropyrimidine, and platinum chemotherapy as first-line therapy for stage IV HER2-positive metastatic gastroesophageal cancer. Janjigian and colleagues recently reported in abstract form initial results from NCT02954536 [60]. Patients with previously untreated HER2 IHC3+ or FISH+ tumors irrespective of PD-L1 status were treated with pembrolizumab 200 mg, trastuzumab 6 mg/kg (after 8 mg/kg load), oxaliplatin 130 mg/m^2^ every 3 weeks, and capecitabine 850 mg/m^2^ dosed 2 weeks on/1 week off (or 5-FU). The ORR was 83% (17 PRs and 3 CRs) with a median PFS of 11.4 months and median OS not reached at the time of data analysis [60]. Interestingly, 56% of the pre-treatment tumors demonstrated detectable *HER2* gene amplification by NGS, with the remainder of the HER2-overexpressing tumors being negative by NGS, again reflecting the high degree of HER2 intratumoral heterogeneity that exists in this disease. In attempts to validate this combination approach in HER2-targeted first-line therapy, the ongoing phase 3 KEYNOTE-811 trial (NCT03615326) is randomizing patients with advanced HER2-positive gastric or GEJ adenocarcinoma to fluoropyrimidine, platinum, and trastuzumab chemotherapy with or without the addition of pembrolizumab. If ultimately larger datasets such as the KEYNOTE-811 trial demonstrate that augmenting immune targeting of the HER2 receptor is what improves the paradigm for first-line therapy, this may call into question whether disruption of HER2 signaling is necessary against HER2-positive gastroesophageal cancer. While such a hypothesis remains a point of conjecture until future data emerges, this may account for the failures of lapatinib and pertuzumab where these agents act primarily through inhibition of HER2 signaling.

Future efforts to augment immune approaches include genetically modified T cells with reprogrammed, recombinant chimeric antigen receptors, or CAR-T cells, which can target tumor cells expressing specific surface antigens without major histocompatibility complex (MHC) restriction to eliminate them [61]. CAR-T cells targeting CD19 have entered into the clinic for B cell malignancies, and engineering of CAR-T cells against solid tumor antigens have been both promising and challenging. Initial trials with CAR-T cells targeting HER2 demonstrated fatal toxicity in the first treated patient, which appeared mediated by recognition of the low density of HER2 receptors expressed in normal lung epithelium resulting in severe cytokine release and pulmonary failure [62]. The newer generation of CAR-T cells targeting HER2 with lower affinity has demonstrated acceptable safety to date in an initial trial of HER2-positive sarcoma patients [63].

Natural killer (NK) cells are important cytotoxic lymphocytes in innate immunity with similar cytolytic activity as cytotoxic T cells, but they do not need recognition and engagement of the major histocompatibility complex (MHC) on target cells. Thus, they can be advantageous in killing tumor cells which have lost MHC expression to escape T cell surveillance. NK cells also express Fc receptors to recruit antibody-dependent cellular cytotoxicity (ADCC). FATE-NK100 is an NK cell product that uses ex vivo activated effector cells harboring enhanced anti-tumor activity. An ongoing trial (NCT03319459) is testing FATE-NK100 in combination with trastuzumab in subjects with HER2-positive advanced breast and gastric cancer, as well as other advanced HER2-positive solid tumors.

## Conclusion

Discernment of metastatic gastroesophageal cancer patients with tumor HER2 overexpression remains of significance in improving treatment outcomes. However, to enable progress beyond currently approved therapies in this molecular subset will require composite testing strategies to properly capture spatial and temporal tumoral heterogeneity that will enhance precision medicine efforts. The advent of targeted NGS in analyzing both tumor and ctDNA has yielded enlightening data that may have immediate applicability in the clinic. Intelligent incorporation of these biomarkers can improve the therapeutic impact of the next generation of HER2-targeted trials in gastroesophageal cancer.

## Abbreviations

AACR: American Association for Cancer Research
ACRG: The Asian Cancer Research Group
ADCC: Antibody-dependent cellular cytotoxicity
ASCO: American Society of Clinical Oncology
ASCP: American Society for Clinical Pathology
CAP: College of American Pathologists
CAR-T: Chimeric antigen receptor (CAR)-T cells
CEP17: Centromeric region of chromosome 17
cfHER2amp: Circulating HER2 gene amplification
CI: Confidence interval
ctDNA: Circulating tumor DNA
ECD: Extracellular domain
EGFR: Epidermal growth factor receptor
FcR: Fc receptors
FISH: Fluorescence in situ hybridization
GC: Gastric body adenocarcinoma
GEA: Gastroesophageal adenocarcinomas
GEJ: Gastroesophageal junction
HER2: Human epidermal growth factor 2
HER2amp: HER2-amplified
HR: Hazard ratio
IHC: Immunohistochemistry
MHC: Major histocompatibility complex
MTD: Maximum tolerated dose
NGS: Next-generation sequencing
NK cell: Natural killer cell
ORR: Overall response rate
OS: Overall survival
PD-1: Program death 1
PD-L1: Programmed death-ligand 1
PDX: Patient-derived xenograft
PFS: Progression-free survival
RP2D: Recommended phase 2 dose
TCGA: The Cancer Genome Atlas
TCR: T cell antigen receptor
T-DM1: Trastuzumab emtansine
TKI: Tyrosine kinase inhibitors
TLR: Toll-like receptor
ToGA: Trastuzumab for Gastric Cancer
US: United States
VEGFR: Vascular endothelial growth factor receptor
